# Stress and Mental Health in Families With Different Income Levels: A Strategy to Collect Multi-Actor Data

**DOI:** 10.2196/resprot.2832

**Published:** 2014-01-02

**Authors:** Koen Ponnet, Edwin Wouters

**Affiliations:** ^1^Department of Sociology, University of AntwerpAntwerpBelgium; ^2^Higher Institute for Family SciencesHuBrusselBrusselsBelgium

**Keywords:** stress, mental health, well-being, family process, multi-actor approach

## Abstract

**Background:**

Several studies have focused on family stress processes, examining the association between various sources of stress and the mental health and well-being of parents and adolescents. The majority of these studies take the individual as the unit of analysis. Multi-actor panel data make it possible to examine the dynamics of the family context over time and the differentiating effects of individual roles within the same family. Accurate information about family processes allows practitioners to provide support that enhances family resilience and minimizes the risk of mental health problems.

**Objective:**

Our study contributes to the research on family stress processes by focusing on families with different income levels, and by collecting panel data from mothers, fathers, and adolescents within the same family.

**Methods:**

The relationship between mothers, fathers, and children (RMFC) study is an ongoing Flemish multi-actor panel study that aims to enhance our understanding of family processes that protect the mental health and well-being of two-parent families with a target adolescent between 11 and 17 years old. Mothers, fathers, and children provide information about various aspects of family life, including finances, sources of stress, health, mental health, parenting, and coping strategies. Measures have been chosen whenever possible that have sound conceptual underpinnings and robust psychometric properties. The study posed two challenges. First, economically disadvantaged families are difficult to reach. Second, the collection of multi-actor data is often plagued by high nonresponse. To ensure that the families were targeted as successfully as possible, the study employed a purposive nonprobability sampling method.

**Results:**

The RMFC study is one of the largest triadic panel studies of its kind. The first wave of quantitative data collection was conducted between February 2012 and January 2013. A total of 2566 individuals of 880 families participated in our study. The second wave of data collection will be undertaken 6-12 months later.

**Conclusions:**

The strength of the RMFC study is its multi-actor panel approach of data collection among families with different income levels. Strategies that were followed to address the empirical issues involved with the sampling design are discussed, together with theoretical and practical implications.

## Introduction

### Background

Growing up and living with financial hardship is detrimental to one’s physical and mental health. Rates of psychopathology and various types of mental disorders (eg, depression, anxiety) are higher among individuals from low-income families than among individuals from middle- and high-income families [1,2]. Financial hardship creates a context of stress in which stressors build on one another and contribute to mental health problems for adults and children [3]. In addition, children from low-income families are more likely to engage in problematic behavior, such as aggressive behavior and substance abuse [4]. Most research on the negative influence of financial hardship or stress on families and children has been based on the family stress model [5,6]. This model specifies that high levels of financial stress have detrimental effects on parental psychopathology, interparental conflict, and parenting, and these parental problems damage children’s mental health and well-being.

Despite strong support for the original and expanded family stress models (eg, including social support, health problems) in a variety of contexts, most studies use data from one family member to examine relationships between family members [7]. Our study expands upon previous studies on stress processes by including paired data from both parents and an adolescent. Given the lack of extensive research using a dyadic approach to consider family stress processes [8], research that takes into account the interdependence and mutual influence between mothers and fathers—or between parents and adolescents—could improve our understanding of the processes that protect the mental health and well-being of parents and adolescents. This understanding is essential for developing and implementing successful intervention programs. Accurate information about the mechanisms of stress allows practitioners to provide support that enhances family resilience and minimizes the risk of mental health problems.

Our paper describes how the dyadic approach to data collection and analysis differs from the more common individualistic approach. This is followed by a description of the aims and study design of a project entitled, “Relationships between mothers, fathers, and children” (RMFC). The unique contribution of the interuniversity RMFC project is its multi-actor panel approach to data collection: several types of information (eg, finances, various sources of stress, health, mental health, parenting, and coping strategies) were collected from mothers, fathers, and adolescents within the same families. The first wave of data collection started in February 2012 and ended in January 2013. The second wave of data collection will be undertaken 6-12 months later. The outcomes will include a detailed picture of family functioning and an enhanced understanding of processes that protect the mental health and well-being of both parents and adolescents.

### Individualistic Versus Dyadic Approaches

One common shortcoming of many studies on family stress processes is that they focus on either mothers or fathers. Because mothers and fathers belong to the same family, however, they should not be viewed simply as two independent individuals. They share a characteristic known as nonindependence [9]. The characteristic of nonindependence can be assumed if two scores from two members of a dyad are more similar to one another than are two scores from two people who are not members of the same dyad. Information about nonindependence has theoretical and statistical implications. Theoretically, nonindependence can be used to infer reciprocity, synchrony, or influence within a dyad. Statistically, it requires that the data be analysed in ways that include both the dyad and the person as units of analysis. If it is ignored, nonindependence can bias tests of significance [9,10].

By administering data from two parents within the same family, both the person and the dyad can be used as units of analysis. The choice to focus on the person or the dyad in various constructs is related to actor-partner effects within the dyads [11]. An actor effect refers to the impact of an independent variable of a person on an outcome variable of the same person (eg, a mother who experiences high levels of financial stress is more likely to experience depressive feelings). A partner effect occurs when a person’s score on an independent variable affects the partner’s score on an outcome variable (eg, increased levels of stress experienced by one parent might be negatively associated with the partner’s marital satisfaction). As such, the use of a dyadic approach enables researchers to study separate paths through which financial stress experienced by mothers and fathers affects depressive symptoms, health, marital problems, or parenting behaviors of both the person and the partner.

The dyadic approach to data collection and analysis is nevertheless not limited to mother-father dyads. Mother-adolescent or father-adolescent dyads can also be used as unit of analysis. Although several researchers advocate the use of multiple informants in studies on family functioning [12], the tendency to consider children or adolescents as active agents is quite recent [13]. Epstein and colleagues [14] described three reasons for considering the perceptions of multiple family members (ie, mothers, fathers, and adolescents) in the assessment of families. First, each family informant provides a unique perspective on events occurring within their families. Second, different family members may provide slightly different information based on their own experiences in the family and on differential knowledge about the others. Third, although family members can witness the overt behaviors of themselves and other family members, they can be aware of only their own internal states and perceptions.

We strongly believe that inclusion of multiple family members in studies on family stress processes could enhance knowledge concerning their mutual influence. For this reason, the RMFC project (as outlined below) applied a multi-actor panel design, including information on both of the married or cohabiting parents, as well as on a target adolescent between 11 and 17 years of age.

### Aims

The overall aim of the RMFC project is to explore how various sources of stress affect the mental health and well-being of parents and adolescents. Our study contributes to previous research on family stress processes in several ways.

First, most previous research studies on family stress processes were conducted in the United States. Our study was conducted in the Dutch-speaking part of Belgium (Flanders), and it should be seen in this context. Because Belgium is quite different from the United States in terms of economic and social security, the experiences and responses of families might differ.

Second, the RMFC study focuses on low-, middle-, and high-income families. Although financial stress and ongoing strains seem to be more prevalent in low-income families compared to middle- or high-income families, it appears that low-income families are also more vulnerable to events and strains (ie, different sources of stress have more devastating impact in these families) [4]. An important issue is how to understand the processes that are responsible for the variability that exists among families with different income levels. This includes the identification of factors that cause some families, or family members, to experience mental health problems whereas other families seem not to be compromised. To do so, we collected information on various sources of stress (eg, financial stress, parental stress, marital stress, and daily stress) and coping strategies to manage that stress.

Third, the RMFC project is based on a family-system approach (as described above), in which the family is considered as a complex, integrated whole in which individual family members are necessarily interdependent [15,16]. For this reason, data were collected from mothers, fathers, and adolescents. These triadic data make it possible to examine pathways within and between family members.

Fourth, families were invited to take part in a follow-up study. One major advantage of the panel design stems from its ability to compare the same individual at different times, and hence permit within-individual analyses of individual change. From a multi-actor design standpoint, each family member can have a unique trajectory. The trajectories can differ in magnitude (eg, the rate of change can be more steep for mothers than that for fathers) or pattern (eg, change can be linear for mothers and nonlinear for fathers) [17].

## Methods

### Sampling

In general, probability sampling is the preferred approach for scientifically conducted surveys. A probability sample is defined as a sample in which individuals are chosen at random, such that each individual has a calculable, nonzero probability of selection. The RMFC project, however, used a purposively nonprobabilistic sampling design with oversampling of low-income families. The design was selected for two reasons.

First, the RMFC project involved gaining access to economically disadvantaged families, in addition to middle- to high-income families. This posed a challenge, given that many economically disadvantaged families are “hidden” and notoriously difficult to access in a systematic way [18]. In most studies in which the representative household survey is the golden standard for data collection, such hidden population segments are either lost by definition or, at best, grossly underrepresented [19]. Thus, most studies of low-income families use some form of nonprobability sampling in order to recruit participants [20]. The design has also the advantage of being affordable.

Second, multi-actor data are highly valuable for investigating questions about family functioning, and they improve the reliability of information on the subjective characteristics of household members. Nevertheless, the collection of such data is often plagued by high nonresponse [21]. For example, the recent “Divorce in Flanders” study, in which the sample was drawn from the Belgian National Register, applied a multi-actor design, including information on both currently and formerly married partners, as well as on their children aged 10 years or older. As noted by the researchers [22], the response rate for dyadic data (ie, both mother and father responded to the questionnaire) from married families was 31.41%, while the response rate for triadic data from married families (ie, mother, father, and a child responded to the questionnaire) was 12.75%. This made it difficult to generalize the triadic findings. One of the problems associated with a multi-actor approach is that data collection is complicated by nonresponse on the part of one family member. Whether a particular family member will respond depends upon individual characteristics, in addition to characteristics of the mutual relationships between all family members involved. In a study on nonresponse by secondary respondents in multi-actor surveys, Kalmijn and Liefbroer [21] reported that a parent is more likely to grant permission to collect data from the child if the relationship between the parent and the child is intensive and of good quality. The quality of the relationship also has a positive effect on the likelihood that children will return the questionnaire [21]. Relationship quality thus has an impact on the response process, regardless of the sampling design that is selected.

Taken together, given the lack of a sampling frame for our target population of families, a random selection from the study population was not a realistic option. As recommended by some authors [23,24], however, the RMFC project followed several strategies in order to address the empirical issues involved in the use of a nonprobability sample. More specifically, efforts were made to ensure that the study sample provided adequate statistical power for hypothesis testing. It has been shown that, other things being equal, large samples always produce estimates about true population parameters that are more efficient and unbiased than are those produced by small samples. Furthermore, the researchers engaged in multi-agency collaboration. Finally, a national sample, the European Union Statistics on Income and Living Conditions (EU-SILC) [25], was used to compare our data. Because the purpose of the EU-SILC is different from the purpose of our study, it was possible to use probability sampling.

### Calculation of A Priori Sample Size

The power of a statistical test depends upon the following parameters: the reliability of the sample results, the sample size, the effect size, and the significance criterion. Following the proposed conventions described by Cohen [26], we adopted a desired power value of at least .80 and a desired alpha score of no greater than .05. Based on previous multi-actor research studies on family stress processes [7,27] and taking into account the number of measures that we wanted to include in our future family stress models (see below), we expect to study structural equation models with a maximum of 22 observed and 8 latent variables. Using the statistical program of Soper [28], the calculation of a priori sample size (with an anticipated medium effect size of 0.3) returned a recommended minimum sample size of 241 households. A more demanding effect size (ie, 0.1) would require us to recruit 625 households.

### Recruitment

Two-parent families with a target adolescent in secondary school (ie, between 11 and 17 years of age) were recruited from February 2012 through January 2013. Families were recruited from five provinces of the Dutch-speaking part of Belgium (ie, Flanders), with assistance from undergraduate students from two institutes of higher education: the Higher Institute for Family Sciences and the University of Antwerp. A two-stage strategy was used to reach the households. First, each of the students from the Higher Institute for Family Sciences (n=85) was instructed to recruit low-, middle-, and high-income two-parent families. Students received course credit for their recruitment efforts. The average age of the students from the Higher Institute was 34.85 (SD 1.24) and most were working in the social services. As such, the project took advantage of the social networks of the students in order to obtain a large set of potential respondents. Each of the targeted families (mother, father, and target adolescent) was sent a letter explaining the purpose of the research. The families were subsequently contacted and asked to participate. In total, 1020 packages of envelopes and questionnaires were distributed (12 per student), and 824/1020 (80.78%) were returned by post. Second, four 21-year-old students from the University of Antwerp recruited 56 low-income families through community agencies, including centers for general welfare (CAW) and public centers for social welfare (OCMW), as well as through service and meeting centers. The students contacted 25 community agencies distributed across the different regions, and 14 volunteered to cooperate. Personnel in the community agencies selected potential families, and the students contacted them to assess their willingness to participate. Once they agreed, families were given the packages of envelopes and questionnaires.

### Ethics and Data Collection

Each participant received a plain-language statement and a written informed-consent form. The study protocol was approved by the Ethics Committee of the University of Antwerp (Belgian registration number: B300201215397).

Each family received a package of three envelopes and questionnaires. A letter accompanying the questionnaire introduced the study as an investigation of “the relationship between mothers, fathers, and children” and provided information on the purpose of the study in lay terms. The first page of the questionnaire instructed the target participants to complete the booklets individually and not to discuss the content of the questionnaire with one another. The booklets were to be returned in a stamped envelope. Mothers, fathers, and adolescents were asked to sign written consent forms, which were to be returned by post in a separate envelope. All families were also asked if they were willing to take part in future research. It was made clear in the written informed-consent form that participation was voluntary. In total, 51.2% (418/817) families of the triads volunteered to be followed up.

### Content of the Parent Questionnaire

The questionnaires for mothers and fathers were identical (except for such phrasings as “he/she” or “father/mother”) and contained 290 items. A small pilot study (six mothers and fathers) revealed that it took about 40 minutes to complete the parent survey.

The questionnaire included items on sociodemographic indicators, including age, education, nationality, country of origin, religiosity, occupation, civil status, length of relationship, number of household members, and, in the case of multiple children, the age of youngest and oldest child in the household. Parents were also asked to provide sociodemographic information on the target adolescent, including the age and gender of the adolescent, relationship to the adolescent (eg, biological mother, stepmother), education, school years repeated, and the presence of any developmental disorders.

To gain insights into various aspects of family functioning, measures have been chosen whenever possible that have sound conceptual underpinnings and robust psychometric properties. To assess parental mental health, the Hospital Anxiety and Depression Scale [29] and a short form of the CES-D [30] were included. The physical health item was drawn from the EU-SILC instrument [31]. Interparental relationship was measured using the O’Leary-Porter Scale [32], subscales from the Conflicts and Problem-Solving Strategies questionnaire [33], the Multidimensional Stress Questionnaire for Couples [34], and the Quality of Marriage Index [35]. Parent-adolescent relationship was assessed using the Parent-Adolescent Communication Scale developed by Barnes and Olson [36], subscales from the Parental Behavior Scale [37], and the Psychological Control Scale [38]. The questionnaire also included subscales from the Dutch version of the Parenting Stress Index [39] and the Parenting Sense-of-Competence Scale [40]. Information about the family’s financial situation was assessed with self-constructed items on savings, financial stress, financial insecurity and financial needs, as well as with items drawn from the EU-SILC [31]. Consistent with other studies involving fragile families [41,42], items on coping strategies and social support were included as well, like the Carver Coping Scale [43]. Finally, the questionnaire included items about the adolescent’s school competence, and the adolescent’s emotional and behavioral problems were assessed using the Child Behavioral Checklist [44].

### Content of the Adolescent Questionnaire

The adolescent questionnaire contained 191 items and took about 25 minutes to complete. Sociodemographic questions included gender, age, number of brothers and sisters, education, and the marital status of parents. Information on stress was assessed with items drawn from the Sources of Stress Index [45]. Adolescents completed scales on parent-adolescent relationship twice, once for the mother-child relationship and once for the father-child relationship. Scales included were the Parent-Adolescent Communication Scale developed by Barnes and Olson [36], subscales from the Parental Behavior Scale [37], and the Psychological Control Scale [38]. Peer attachment was assessed with a subscale from the Inventory of Parent and Peer Attachment [46]. Finally, similar to the parent questionnaire but adapted to the adolescent perspective, items were included about adolescents’ school competence, coping strategies, and the adolescent’s emotional and behavioral problems.

## Results

### Sample Characteristics

Over the 12-month survey period, 880 households were recruited: 824 households in the first stage and 56 households in the second stage (see the above-mentioned recruitment procedure). The dataset contained information on 817 triads (mother, father, and adolescent) and 857 mother-father dyads. Table 1 provides an overview of the number of participants. The average ages of fathers and mothers were 46.03 (SD 5.10) and 43.72 (SD 4.56) years, respectively. Within our sample, 2.7% (23/848) of the mothers and 4.0% (34/850) of the fathers had completed preprimary or primary education; 33.7% (286/848) of the mothers and 41.5% (353/850) of the fathers had completed secondary education; and 63.6% (539/848) of the mothers and 54.5% (463/850) of the fathers had completed postsecondary education. With regard to work status, 95.3% (810/850) of the fathers and 84.3% (721/855) of the mothers worked either full-time or part-time. Furthermore, three-person households accounted for 10.4% (89/855) of the sample, four-person households for 46.8% (400/855), five-person households for 29.2% (249/855), six-person household for 9.6% (82/855), and households of seven or more people for 4.1% (35/855). Using the *modified OECD equivalence* scale [47], the average household income of our sample was €1592.95 (SD 604.17).

**Table 1:** Overview of the RMFC dataset (N=2566).

	Households, n (%)	Individuals, n (%)
Triadic data (mother, father, and adolescent)	817 (92.84)	2451 (95.51)
Dyadic data (mother and father)	40 (4.54)	80 (3.12)
Dyadic data (parent and adolescent)	12 (1.36)	24 (0.94)
Individual data (mother or father)	11 (1.25)	11 (0.43)
Total	880	2566

### Comparisons Between the RMFC and EU-SILC Samples

The EU-SILC is the EU reference source for microlevel data on income and living conditions. The dataset includes internationally and cross-temporary comparable variables for all EU Member States [48]. The reference population of the EU-SILC consists of private households residing in the participating countries at the time of selection. In this study, we selected households from the Dutch-speaking part of Belgium that had at least one child between 11 and 17 years of age (317/3084, 10.28%). Calculations are based on the EU-SILC 2011 user database.

**Figure 1:**
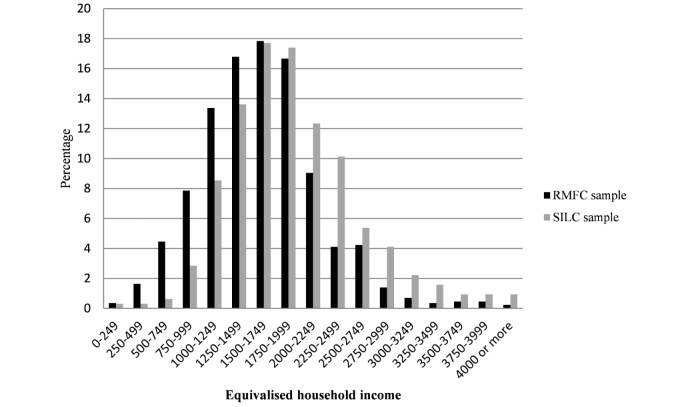
Equivalised household income of the RMFC and the SILC sample.

Our findings revealed that the mean age of the mothers and that of the fathers did not differ significantly between the two samples (*F*_1,1156_=3.25 for mothers and *F*_1,1156_=2.25 for fathers). As shown in Table 2, the educational attainment of mothers in the RMFC sample was somewhat higher than was that of the EU-SILC sample (χ^2^_4_=18.66, *P*<.001). With regard to fathers’ educational attainment, no significant differences were found between the two samples (χ^2^_4_=8.69, *P*=.069). With regard to the employment of parents (Table 2), no significant differences were found between the samples for mothers (χ^2^_4_ *=*4.28, *P*=.369) or for fathers (χ^2^_4_=1.41, *P*=.888). As shown in Table 2, households in the EU-SILC sample were more likely to consist of three members and less likely to consist of five or more members (χ^2^_4_=14.28, *P*=.006). As expected, low-income households were oversampled in the RMFC dataset, relative to the EU-SILC dataset. Figure 1 presents an overview of household income.

**Table 2:** Characteristics of the RMFC and the EU-SILC sample.

		RMFC sample, (n=857)	EU-SILC sample, (n=317)
		n (%)	n (%)
**Educational level of mothers**			
	Preprimary education	12/848 (1.42)	3/317 (0.95)
	Primary education	11/848 (1.30)	8/317 (2.52)
	Lower secondary education	53/848 (6.25)	24/317 (7.57)
	(Upper) secondary education	233/848 (27.48)	122/317 (38.49)
	Postsecondary education	539/848 (63.56)	160/317 (50.47)
**Educational level of fathers**			
	Preprimary education	23/850 (2.71)	3/317 (0.95)
	Primary education	11/850 (1.29)	9/317 (2.84)
	Lower secondary education	99/850 (11.65)	31/317 (9.78)
	(Upper) secondary education	254/850 (29.88)	112/317 (35.33)
	Postsecondary education	463/850 (54.47)	162/317 (51.10)
**Employed**			
	Mothers	721/855 (84.33)	266/317 (83.91)
	Fathers	810/850 (95.29)	296/317 (93.38)
**Household members**			
	Three	89/855 (10.41)	54/317 (17.03)
	Four	400/855 (46.78)	158/317 (49.84)
	Five	249/855 (29.12)	71/317 (22.39)
	Six	82/855 (9.59)	24/317 (7.57)
	Seven or more	35/855 (4.09)	10/317 (3.15)

### An Example of Future Research Directions

**Figure 2:**
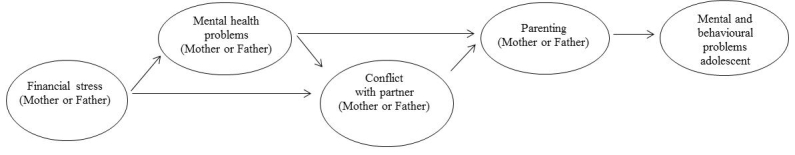
An individual approach of the family stress model.

During the past two decades, a large body of research has focused on family stress processes [49], examining family-based pathways through which financial stress is associated with the adjustment of parents and adolescents. Most research on the negative influence of financial hardship on families and adolescents has been based on the family stress model [6,49]. This model predicts that high levels of financial stress have detrimental effects on parental mental health, interparental conflict, and parenting, and these parental problems damage children’s mental health and well-being (see Figure 2). To date, studies that have applied the family stress model have typically analysed data on mothers and fathers separately [50]. These studies thus neglect the interdependence of the two parents and the mutual influence that they have on each other.

**Figure 3:**
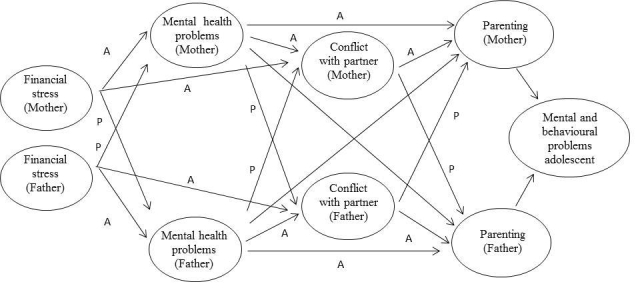
An actor-partner approach of the family stress model. A: actor effects; P: partner effects.

The RMFC study may contribute to the research on family stress processes by its multi-actor approach, which enables us to test more advanced theoretical models. For instance, as shown in Figure 3, analyses can be grounded in the actor-partner interdependence model (APIM) [9], a multi-actor approach which proposes that the predictor variable of both the respondent (actor effects) and the respondent’s partner (partner effects) influence the respondent’s outcome variable [51]. The APIM allows for the testing of both actor and partner effects, and may thus provide better insights into how mothers and fathers each respond to financial stress.

## Discussion

### Principal Findings

We described a strategy to collect multi-actor data from families with different income levels. The families participating in the study were living in the Dutch-speaking part of Belgium. To improve our understanding of processes that protect the mental health and well-being of both parents and adolescents, we collected information about various aspects of family life, including finances, stress, health, mental health, parenting, and coping strategies.

Gaining access to economically disadvantaged families and recruiting mothers, fathers, and adolescents to participate in a research study poses two challenges. As noted above, it would be impossible to obtain a random sample of the study population, given the absence of a comprehensive population list. The RMFC project therefore employed a nonprobability sampling method, purposive sampling, in order to ensure that this group was targeted as successfully as possible. One major drawback of purposive sampling is that it limits the ability to generalize results. To mitigate this problem, the researchers attempted to obtain a large sample size, and they engaged in multi-agency research collaborations. A posteriori comparisons between the RMFC sample and the EU-SILC probability sample revealed more similarities than differences between the demographic characteristics of the families in the two samples. For all of these reasons, the present nonprobability sampling procedure can be considered as an alternative or as a complementary strategy for attaining more comprehensive data with which to investigate research questions concerning family stress processes.

### Conclusions

Multi-actor information on family functioning has both theoretical and practical implications. For example, one limitation in the current literature that can be overcome by researchers using the RMFC data involves the relative lack of attention to possible gender differences in the pathways from stress to parenting [52,53]. This limitation stems from the fact that early parenting research focused almost exclusively on mothers, partly due to the common assumption that mothers play a central role in child development [54].

By focusing on the dyad as unit of analysis, researchers can examine effects within and between parents and begin to understand the dynamic processes that constitute the relationship [17]. The multi-actor panel approach will make it possible to examine the dynamics of the family context over time and the differentiating effects of individual roles within the same family. In this manner, the study will provide better insight into differences in the ways in which family members respond to different sources of stress. This knowledge might subsequently help practitioners in their efforts to support fragile families. When the coping strategies are identified and matched to particular stressors and characteristics of the family members, practitioners may then teach the family members to use the strategies that best align to their particular situation and characteristics.

## Abbreviations

APIM: actor-partner interdependence model
EU-SILC: European Union Statistics on Income and Living Conditions
RMFC: relationship between mothers, fathers, and children
